# A rare co-occurrence of hidradenocarcinoma and squamous cell carcinoma in situ on the left posterior calf treated with Mohs micrographic surgery

**DOI:** 10.1016/j.jdcr.2026.01.008

**Published:** 2026-01-19

**Authors:** Randy W. Head, Conner M. Bacon, Richard Jahan-Tigh, Caitlin G. Robinson

**Affiliations:** a LSU Health Sciences Center School of Medicine, New Orleans, Louisiana; b Medical Pathology Associates, Houston, Texas; c Louisiana Mohs and Skin Surgery, Monroe, Louisiana

**Keywords:** adnexal tumor, collision tumor, dermatopathology, eccrine sweat glands, hidradenocarcinoma, Mohs micrographic surgery, squamous cell carcinoma in situ

## Introduction

Hidradenocarcinoma is a rare, malignant tumor of eccrine sweat glands, accounting for 6% of eccrine tumors and 0.001% of all tumors.^1^ Cases have been described in a wide range of ages and different anatomic sites, but are most commonly found on the face and extremities.^2^ In a recent study, the current 10-year overall survival rate is estimated to be 60.2%, with a high likelihood of the tumor metastasizing to distant sites.^3^ Diagnosing and distinguishing this cancer from its benign counterpart, hidradenoma, can be challenging for physicians from both a clinical and histologic perspective.^4^ Clinically, the tumor typically presents as a slow-growing, painless, firm nodule that can secrete serosanguineous fluid.^1^ Upon gross examination, these lesions may appear well circumscribed, but the malignant features become clear histologically. Key features include asymmetry and infiltrative growth into the dermis or subcutaneous fat, pleomorphism and necrosis. Additionally, these tumors often contain mixed epithelial cell types, including squamous, mucinous, and clear cells containing glycogen, ductal structures, and cyst formation. At higher magnification, multinodular infiltrative nests of atypical cells support the diagnosis of hidradenocarcinoma.^2^ Currently, there are no established guidelines for the treatment of this tumor.^4^ Most recommendations suggest wide local excision as the primary treatment option.^2^^,^^5^ However, in the right clinical setting, Mohs micrographic surgery could be considered.^5^ In the following case, a rare occurrence of hidradenocarcinoma associated with squamous cell carcinoma in situ (SCCIS) is successfully treated with Mohs micrographic surgery.

### Case

**Fig 1:**
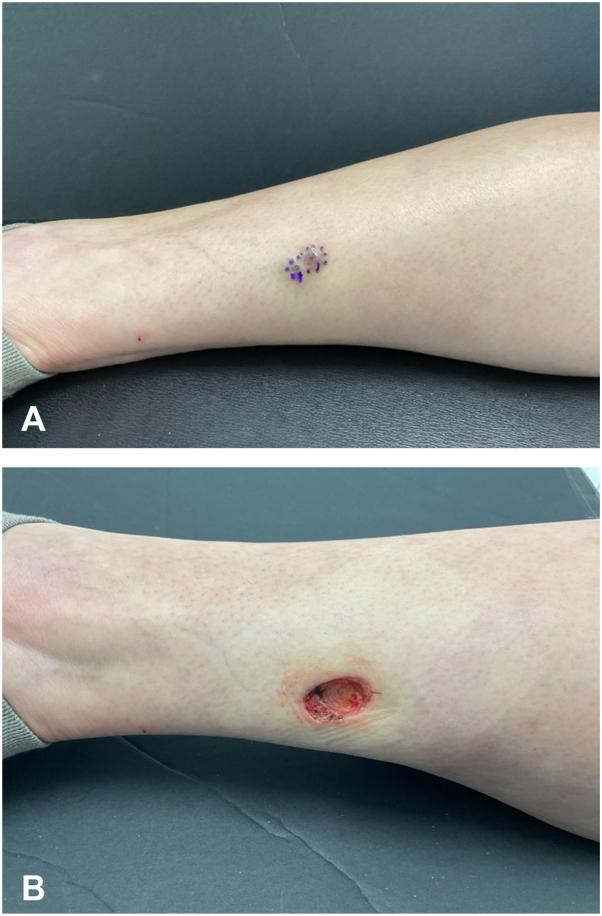
**A,** Marked 1.7 × 1.0 cm lesion prior to first Mohs surgery. **B,** Postoperative excision site with a diameter of 1.9 × 1.2 cm and margins of 0.1 cm.

A 68-year-old female presented for Mohs surgery of a biopsy-proven SCCIS located on the left posterior calf. Upon examination, 2 <1 cm pigmented nodules adjacent to the prior biopsy site were noted (Fig 1, *A*). Various treatment options were discussed for the SCCIS. These treatments included Mohs surgery, excisional surgery, radiation therapy, and various topical therapies. The patient was assigned a Mohs appropriate use criteria score of 6, indicating that additional information and physician judgment were required to determine appropriateness of Mohs surgery. Given the size greater than 1 cm and nodular appearance, it was decided to proceed with Mohs surgery (Fig 1, *B*).

**Fig 2:**
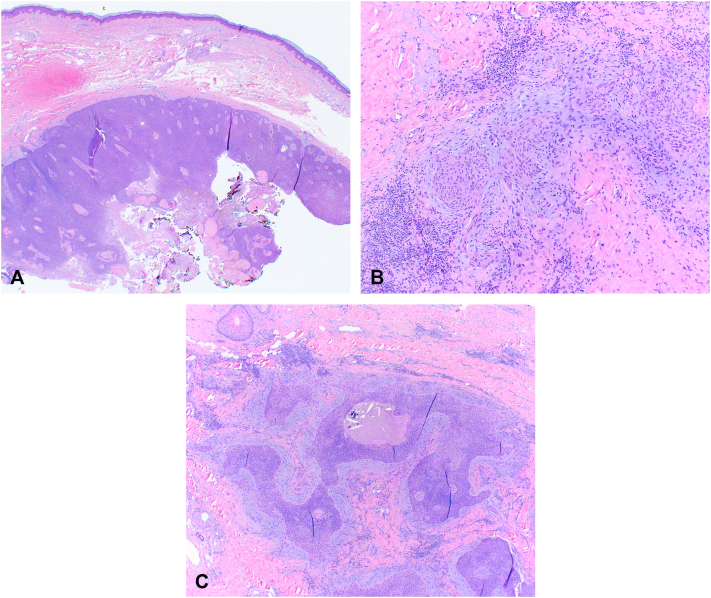
H&E sections show a dermal basaloid neoplasm with ductal differentiation, mucin production, and thickened hyalinized basement membrane material. Some parts of the tumor are cytologically atypical, glassy, and have nuclear pleomorphism. Melanin pigment is scattered throughout the neoplasm. **A,** 20× magnification. **B,** and **C,** 40× magnification. *H&E*, Hematoxylin and eosin.

**Fig 3:**
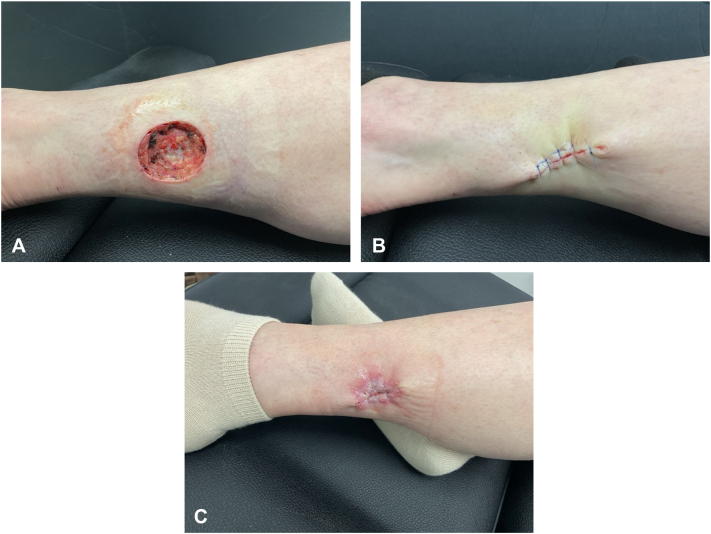
**A,** Final Mohs surgery site with a final defect size of 3.5 × 3.0 cm. **B,** Suture site with intermediate purse string closure. **C,** Postoperative site 2 weeks postsurgery, showing proper healing and granulation.

Due to the presence of the 2 pigmented nodules adjacent to the Mohs site, the lesion was debulked, and the debulked tissue was processed for evaluation along with the first Mohs layer. Upon interpretation of the Mohs slides, both the debulk and the first layer revealed a dermal mass consisting of monomorphic, basaloid cells. Due to the unexpected histology, Mohs surgery was paused, and the specimens (both the central debulk and first Mohs layer) were submitted for permanent sectioning and dermatopathological evaluation. The permanent section pathology resulted as hidradenocarcinoma. The diagnosis was rendered using morphology on hematoxylin and eosin stain. No immunohistochemical stains were used (Fig 2). Because the histology of the lesion was easily recognizable to the Mohs surgeon and debulk histology was available to be viewed for comparison, it was decided to continue with the original plan for Mohs surgery. The following week, Mohs surgery was continued. An initial margin of 0.5 cm was taken around the prior surgical site (Fig 3, *A*). Utilizing hematoxylin and eosin stains, frozen section analysis showed no evidence of residual tumor at the margin after 1 Mohs layer.

Surgical repair was performed using a purse-string intermediate closure due to the inelasticity of the skin as well as the location of the wound in an area of active movement. The final wound length was 3.5 cm (Fig 3, *B*), and the patient was scheduled to return for a wound check and suture removal in 14 days. At the time of suture removal, the wound was well-healed (Fig 3, *C*). At her 1-month follow-up, the patient underwent ultrasound imaging of her inguinal lymph nodes to rule out regional metastasis, which demonstrated no abnormal findings. At the patient’s 6-month follow-up, there was no evidence of recurrence. She is scheduled to return for a 12-month follow-up for her final dermatologic evaluation. The patient will continue long-term surveillance with oncology.

## Discussion

Hidradenocarcinoma is a malignant cutaneous neoplasm derived from the eccrine sweat glands. Sweat gland tumors are challenging to diagnose due to the morphologic overlap with other benign neoplasms.^6^ The incidence is similar between men and women and is most commonly diagnosed in patients between the ages of 50 and 70.^2^ In most cases, patients remain asymptomatic other than general pain, occasional bleeding, and discomfort from the site of the lesion. However, the tumor can metastasize, primarily involving regional lymph nodes, bones, and the skin. Though the literature is limited and cases are exceedingly rare, this tumor has an estimated recurrence rate of 60% and a 50% metastasis rate.^7^

After a review of the literature using the terms “hidradenocarcinoma,” “malignant clear cell hidradenoma,” “SCCIS,” and “Bowen’s disease,” no prior case of hidradenocarcinoma and SCCIS at the same anatomic site was identified. In addition to the rarity of this copresentation, our case highlights the use of Mohs surgery in discovering a more concerning malignant tumor underlying a biopsy-proven SCCIS. Had the lesion been treated with topical therapeutics or superficial destruction alone, the deeper, underlying aggressive malignancy could have been missed. This case demonstrates how important it is for providers to better investigate atypical presentations and shows how beneficial the histological assessment during Mohs surgery is in detecting unexpected neoplasms. Due to their aggressive nature, hidradenocarcinomas require timely diagnosis and treatment. Our findings also demonstrate how Mohs surgery could be considered as primary treatment due to the tumor’s recognizability on frozen sections and lack of need for special stains. A debulk section or review of initial biopsy slides could be useful to review in preparation for Mohs surgery. Further studies are needed to establish efficacy of Mohs surgery for hidradenocarcinoma. This case adds to the literature by documenting a previously unreported co-occurrence of hidradenocarcinoma and SCCIS at the same anatomic site, as well as the advantages of Mohs surgery in detecting and treating these rare presentations.

## Conflicts of interest

None disclosed.
